# RETRACTION: Effects of Comprehensive Nursing Intervention in Patients With Diabetic Foot Ulcers: A Meta‐Analysis

**DOI:** 10.1111/iwj.70272

**Published:** 2025-03-03

## Abstract

**RETRACTION**: FengY.‐L. and YuL. , “Effects of Comprehensive Nursing Intervention in Patients with Diabetic Foot Ulcers: A Meta‐Analysis,” International Wound Journal 21, no. 2 (2024): e14696, 10.1111/iwj.14696.PMC1083112842052960

The above article, published online on 31 January 2024, in Wiley Online Library (http://onlinelibrary.wiley.com/), has been retracted by agreement between the journal Editor in Chief, Professor Keith Harding; and John Wiley & Sons Ltd. Following an investigation by the publisher, all parties have concluded that this article was accepted solely on the basis of a compromised peer review process. The editors have therefore decided to retract the article. The authors did not respond to our notice regarding the retraction.

**RETRACTION**: Y.‐L. Feng and L. Yu , “Effects of Comprehensive Nursing Intervention in Patients with Diabetic Foot Ulcers: A Meta‐Analysis,” International Wound Journal 21, no. 2 (2024): e14696, 10.1111/iwj.14696.PMC1083112842052960

The above article, published online on 31 January 2024, in Wiley Online Library (http://onlinelibrary.wiley.com/), has been retracted by agreement between the journal Editor in Chief, Professor Keith Harding; and John Wiley & Sons Ltd. Following an investigation by the publisher, all parties have concluded that this article was accepted solely on the basis of a compromised peer review process. The editors have therefore decided to retract the article. The authors did not respond to our notice regarding the retraction.

